# Treatment of myasthenia gravis with the method of tonifying spleen and replenishing qi in traditional Chinese medicine: A protocol for systematic review and meta-analysis

**DOI:** 10.1097/MD.0000000000028530

**Published:** 2022-01-21

**Authors:** Li Jiang, Peng Xu, Dongmei Zhang, Jing Lu, Tianying Chang, Yinbin Zhang, Jian Wang

**Affiliations:** a College of Traditional Chinese Medicine, Changchun University of Traditional Chinese Medicine, Changchun, Jilin Province, China; b Brain Disease Center, Affiliated Hospital of Changchun University of Traditional Chinese Medicine, Changchun, Jilin Province, China; c Scientific Research Department of Affiliated Hospital of Changchun University of Traditional Chinese Medicine, Changchun, Jilin Province, China; d Research Center of Traditional Chinese Medicine Affiliated to Changchun University of Traditional Chinese Medicine, Changchun, Jilin Province, China; e Clinical Trial Center of Affiliated Medicine of Changchun University of Traditional Chinese Medicine, Changchun, Jilin Province, China.

**Keywords:** myasthenia gravis, protocol, systematic review, tonifying spleen and replenishing qi, traditional Chinese medicine

## Abstract

**Background:**

Myasthenia gravis (MG) is an autoimmune disease that is associated with the production of autoantibodies. The symptoms of MG are mainly mediated by pathogenic antibodies directed against nicotinic acetylcholine receptors, resulting in a decrease in the number of acetylcholine receptors molecules on the postsynaptic membrane of the neuromuscular junction endplate, leading to clinical symptoms of muscle fatigue and weakness. At present, traditional Chinese medicine (TCM) treatment of MG has a better effect by tonifying spleen and replenishing qi. The purpose of this study was to systematically evaluate the efficacy and safety of TCM therapy for tonifying spleen and replenishing qi in the treatment of myasthenia gravis.

**Methods:**

We searched the following databases from their establishment until December 2021: PubMed, Cochrane Library, EMBASE, Web of Science, Springer, CNKI, Wanfang, China Biomedical Database, China Science and Technology Journal Database, Chinese Knowledge Infrastructure, China Clinical Trial Registry, and Baidu Scholars. The literature search language was limited to Chinese and English, and publication time and status were not limited. Randomized controlled trials (RCTs) were included. Two researchers independently searched and screened the articles, assessed their quality, and used the RevMan 5.4.1 software to perform a meta-analysis of the included literature.

**Results:**

This study compared the main outcome indicators: efficacy rate, recurrence rate, quality of life, and quantitative myasthenia gravis (QMG). Secondary outcome indicators were the clinical absolute score, TCM syndrome score, serum acetylcholine receptor antibody level, and electromyogram low-frequency repetitive nerve stimulation.

**Conclusion:**

This study aimed to evaluate whether the TCM method of tonifying spleen and replenishing qi is effective in the treatment of MG and to provide evidence-based data.

**Ethics and dissemination:**

The protocol of the systematic review did not require ethical approval because it did not involve human subjects. This article will be published in peer-reviewed journals and will be presented at conferences.

## 1 Introduction

Myasthenia gravis (MG) is an autoimmune disease mediated by acetylcholine receptor antibody (AChR-Ab), which is dependent on cellular immunity and complements the transmission of neuromuscular junctions.^[1,2]^ MG lesions mainly involve acetylcholine receptors on the postsynaptic membrane of the neuromuscular junction, resulting in a decrease in the nerve signal transmission function of the motor endplate at the neuromuscular junction.^[3,4]^ Typical symptoms include drooping of the upper face, difficulty in swallowing and chewing, voice hoarseness, difficulty in exhaling, striated muscle fatigue, restricted eye movement but normal pupil adjustment, and severe morning and evening symptoms.^[5]^ Modern medical treatments for MG include cholinesterase inhibitors, adrenal cortex hormones, immunosuppressive agents, plasma exchange, and thymectomy.^[6]^ Cholinesterase inhibitors can improve symptoms, have a short onset time, and are only effective drugs for symptom control. Hormonal drugs are commonly used in the clinical practice. Although immunosuppressants have certain effects, they also have many side effects and are prone to relapse after drug discontinuation. Owing to the complicated etiology of MG, the long course of the disease, and its tendency to recur, it has caused a huge economic and psychological burden on patients, families, and society. Currently, there are no specific medications that can completely cure this disease. In recent years, Chinese medicine has achieved good clinical efficacy in the treatment of myasthenia gravis.

Traditional Chinese medicine (TCM) classifies MG into the Weizheng category. TCM believes that spleen qi nourishes the muscles of the whole body, and weak spleen qi causes weakness of the limbs, weakness of the eyelids, and fatigue. Deng Tietao, a master of TCM, believes that the pathogenesis of this disease is deficiency of the spleen and stomach, and the key to the disease is the sinking of qi deficiency.^[7]^ Therefore, TCM treatment of MG is mostly used to invigorate spleen and qi, and has achieved good clinical results. However, the efficacy and safety of MG treatment have not yet been systematically evaluated. This study aimed to systematically and comprehensively retrieve published systematic reviews and meta-analyses of the randomized controlled trial (RCT) literature provided by Chinese medicine for the treatment of myasthenia gravis using the method of tonifying spleen and replenishing qi, and clarifying the direction of clinical treatment.

## 2 Methods

### 2.1 Protocol registration

The study was conducted following the guidelines of the Preferred Reporting Items for Systematic Review and Meta-analysis Protocol (PRISMA-P).^[8]^ This program has been registered on the INPLASY website (registration number INPLASY2021120053). We strictly followed the preferred reporting items of the systematic review and meta-analysis protocols to complete the systematic review.

### 2.2 Inclusion criteria for study selection

#### 2.2.1 Types of studies

We will include Chinese and English literature of RCTs reporting the safety and efficacy of tonifying spleen and replenishing qi in the treatment of myasthenia gravis. Non-RCTs, retrospective research literature, conference abstracts, case reports, repeated publications, and literature without data information were excluded.

#### 2.2.2 Types of participants

All patients should be clearly diagnosed with MG according to internationally recognized diagnostic criteria. The patients were not limited by age, sex, occupation, education, country, ethnicity, or source. Patients with severe cardiovascular diseases, mental illnesses, or an inability to cooperate with treatment were excluded.

#### 2.2.3 Types of interventions

The control group was routinely administered Western medicine treatment, including cholinesterase inhibitors, glucocorticoids, immunosuppressants, alone or in combination, or a placebo.

Based on western medicine treatment in the control group, the intervention group was treated with the method of tonifying spleen and replenishing qi (oral Chinese herbal medicine or Chinese patent medicine, etc), and the dosage form, dosage, and treatment course of the method of tonifying spleen and replenishing qi were not restricted.

### 2.3 Outcome indicator

#### 2.3.1 Main outcome measures

We compared the efficacy rate (using recognized clinical efficacy evaluation standards), recurrence rate, quality of life (assessed using the Business Quality of Life Scale), and QMG scores as the main outcome indicators.

#### 2.3.2 Secondary outcome measure

We used the clinical absolute score (evaluated according to the calculation standard obtained from the Chinese expert consensus on the diagnosis and treatment of myasthenia gravis), TCM syndrome score scale, level of serum AchR-Ab, and results of EMG low-frequency repetitive nerve electrical stimulation as secondary outcome indicators.

### 2.4 Data sources and search strategies

We searched the following databases: PubMed, Cochrane Library, EMBASE, Web of Science, Springer, China Knowledge Network (CNKI), Wanfang, China Biomedical Database, China Science and Technology Journal Database, China Knowledge Infrastructure, China Clinical Trial Registry, and Baidu Scholars. All RCTs on the Chinese medicine method of tonifying spleen and replenishing qi to treat MG were collected. We only searched for articles published in the self-built database until December 2021 in Chinese and English, and did not include unpublished articles. The search terms were as follows: myasthenia gravis, myasthenia, weizheng, tonifying spleen and replenishing qi, tonifying spleen qi, spleen, qi, TCM, Chinese herbal medicine, herbal medicine, Chinese patent medicine, RCTs, controlled clinical trials, and RCTs. Table 1 presents the retrieval strategy using PubMed as an example.

**Table 1:** Search strategy of PubMed database.

Number	Search terms
#1	Myasthenia gravis [Title/abstract]
#2	Myasthenia [Title/abstract]
#3	Weizheng [Title/abstract]
#4	#1 or #2 or #3
#5	Tonifying spleen and replenishing qi [Title/abstract]
#6	Tonifying spleen qi [Title/abstract]
#7	Spleen [Title/abstract]
#8	Qi [Title/abstract]
#9	#5 or #6 or #7 or #8 or #9
#10	#4 and #9
#11	Traditional Chinese medicine [Title/abstract]
#12	Chinese herbal medicine [Title/abstract]
#13	Herbal medicine [Title/abstract]
#14	Chinese patent medicine [Title/abstract]
#15	#11 or #12 or #13 or #14
#16	Randomized controlled trial [Title/abstract]
#17	Controlled clinical trial [Title/abstract]
#18	RCT [Title/abstract]
#19	Randomly [Title/abstract]
#20	#16 or #17 or #18 or #19
#21	#15 and #20
#22	#10 and #21

### 2.5 Data screening and extraction

#### 2.5.1 Study selection

**Figure 1:**
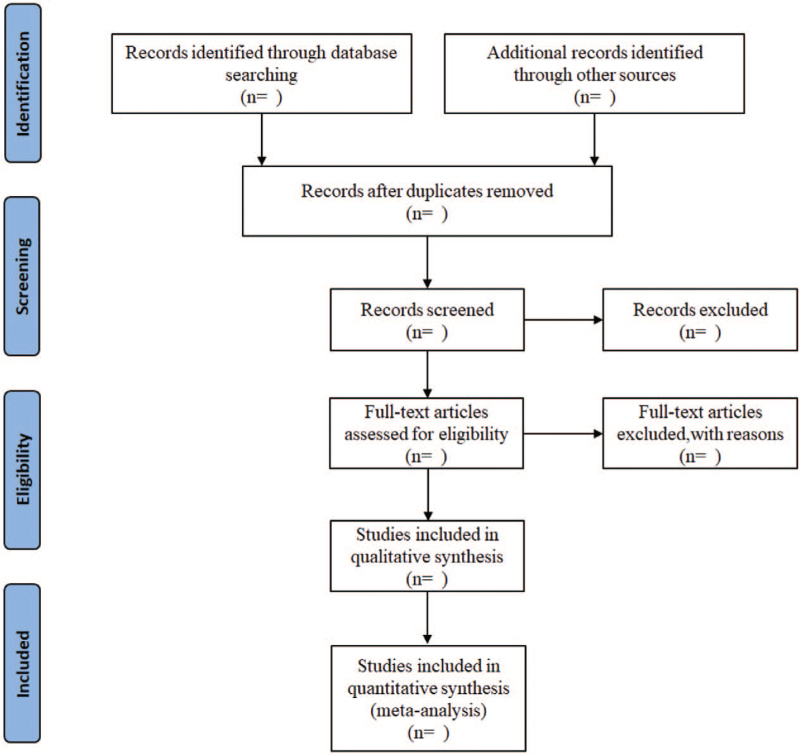
Flow diagram of study selection process.

Two research members (LJ and ZYB) initially screened the articles based on the above inclusion criteria, imported them into EndNote V. 9.0, grouped them, and checked for duplication. The selected documents were then eliminated according to their title and abstract, and the documents were screened. Finally, the third research member (CTY) checks all the documents and makes a final decision based on the standards. All the screening processes were performed independently. If two research members disagree, a third research member will make the decision. We also recorded the excluded documents and explained the reasons for their exclusion. The research screening process is represented by a PRISMA flowchart^[9]^ (Fig. 1).

#### 2.5.2 Data extraction

After completing the literature search, two research members (XP and ZYB) extracted key information from the included literature into an Excel table. The following information was extracted: first author of the literature, year of publication, sex, age, disease course, and sample size of the subject; intervention measures for the intervention and control groups; effective rate, recurrence rate, quality of life score, QMG score, clinical absolute score before and after treatment, TCM syndrome score, serum AchR-Ab concentration, and repetitive nerve stimulation results. Two research members will extract data independently, and if there is a disagreement, they will be discussed, and a third research member (CTY) will decide. When information required for research in the literature was missing, the author was contacted to request the corresponding data.

### 2.6 Assessment of risk of bias in included studies

The methodological quality of RCTs will be assessed independently by two research members (XP and LJ) using the Cochrane risk assessment tool to assess the risk of bias.^[10]^ The following seven criteria will be assessed: random sequence generation, allocation concealment, blinding of participants and personnel, blinding of the result evaluator, incomplete result data, selective reports, and other deviations. The evaluation of low deviation risk, unclear deviation risk, and high deviation risk was given item by item. If the evaluation results of the two research members disagree, the final evaluation result is decided by a third research member (ZDM).

### 2.7 Statistical analysis

We used RevMan V.5.4.1 statistical software to conduct a meta-analysis of the literature, and it was considered statistically significant when *P* < .05. Two research members (XP and JL) performed data extraction, entry, and calculation, and a third research member (ZDM) was responsible for the data verification. We used the mean deviation or standard mean deviation with a 95% confidence interval as the effect measure for the continuous data. Dichotomous results were analyzed using hazard ratios and 95% confidence intervals. *I*^2^ statistics were used to detect clinical heterogeneity. Heterogeneity will be assessed using the chi-square test and Higgins *I*^2^ test. If there was no significant heterogeneity (*I*^2^ ≤ 50%, *P* > .10), a fixed-effects model was used; if *P* < .10, *I*^2^ > 50%, a random-effects model was used for the meta-analysis.

### 2.8 Sensitivity analysis

If the results showed high heterogeneity (*I*^2^ test >50%), sensitivity analysis was performed to obtain stable research results.

### 2.9 Subgroup analysis

If the research data are sufficient, subgroup analysis will be carried out from the following aspects: MG classification, treatment time, and type of Western medicine, to explain the heterogeneity between the studies.

### 2.10 Reporting bias analysis

We analyzed the quality of publication bias using RevMan 5.4.1 software in inverted funnel plots and performed Egger's test when more than 10 trials were included in the meta-analysis.

### 2.11 Grading the quality of evidence

We will use the Grading of Recommendations Assessment, Development, and Evaluation to assess confidence in cumulative evidence.^[11]^ The risk of publication, heterogeneity, indirectness, imprecision, and publication bias was assessed, and the results were divided into four levels: high, moderate, low, and very low.

### 2.12 Ethics and dissemination

In this study, no individual data from participants were involved and ethics approval was not required. This systematic review has been published in a peer-reviewed journal.

## 3 Discussion

MG is an autoimmune disease with an annual incidence of 8 to 10 cases per million and a prevalence of 150 to 250 cases per million. The overall analysis of the age of disease found that the prevalence of MG in women was higher than that in men, with a ratio of approximately 3:2.^[12,13]^ The clinical manifestation of MG is skeletal muscle weakness caused by antibodies, with symptoms ranging from simple ocular symptoms to severe weakness of the limbs, bulbar, and respiratory muscles.^[14]^ Western medical treatment of MG mostly uses anticholinesterase drugs to improve symptoms; however, this drug is only a symptom-controlling drug with a short onset time. Although hormonal drugs and immunosuppressants commonly used clinically have certain effects, MG is still difficult to cure, with large side effects; some adverse reactions will occur, and relapse can easily occur after discontinuing the drug.^[15]^ Long-term clinical practice has proven that TCM can regulate immune function, effectively relieve clinical symptoms, reduce the recurrence of illness, and alleviate the toxicity and side effects of some Western medicines. In recent years, many studies on the treatment of MG with TCM have been published, especially on the treatment of patients with spleen and qi deficiencies by tonifying spleen and replenishing qi. The theory of TCM believes that spleen is the foundation of the acquired nature and is located in the middle Jiao. It produces and transmits qi, spleen governs movement and transformation, and the limbs and muscles. It is a source for qi and blood biochemistry. Insufficient lifting and lowering, insufficient qi and blood metaplasia, loss of muscle nutrition resulting in muscle weakness, and no use of limbs. It is necessary to conduct a systematic review to establish convincing evidence for evaluating the effectiveness and safety of TCM therapies for MG. Therefore, we will adopt a more rigorous systematic evaluation method to provide evidence-based data for the treatment of MG with the method of tonifying spleen and replenishing qi in TCM, and provide new ideas and methods for the treatment and research of MG.

## Author contributions

**Conceptualization:** Li Jiang, Jian Wang.

**Data curation:** Peng Xu, Yibin Zhang.

**Formal analysis:** Peng Xu, Jing Lu.

**Funding acquisition:** Peng Xu, Dongmei Zhang.

**Investigation:** Jing Lu, Yibin Zhang.

**Methodology:** Li Jiang, Tianying Chang.

**Project administration:** Li Jiang, Dongmei Zhang.

**Resources:** Jing Lu, Tianying Chang.

**Software:** Li Jiang, Jing Lu.

**Supervision:** Jian Wang.

**Validation:** Dongmei Zhang, Tianying Chang.

**Visualization:** Peng Xu, Yibin Zhang.

**Writing – original draft:** Li Jiang, Peng Xu.

**Writing – review & editing:** Li Jiang, Dongmei Zhang, Jian Wang.
